# Flow in screen-based gamified virtual reality sports: development and validation of a behaviorally anchored proxy rating scale for adolescents with autism spectrum disorder

**DOI:** 10.3389/fpubh.2026.1947117

**Published:** 2026-09-10

**Authors:** Ahra Oh, Daekeun Kwon

**Affiliations:** 1 Institute of Sport Science, Korea National Sport University, Seoul, Songpa-gu, Republic of Korea; 2 Institute of Sports Health Science, Sunmoon University, Chungnam, Asan, Republic of Korea

**Keywords:** adolescents with autism spectrum disorder, behaviorally anchored rating scale flow, gamification, proxy assessment, scale development, screen-based virtual reality sports, adapted physical activity

## Abstract

**Background:**

Adolescents with autism spectrum disorder (ASD) face substantial barriers to physical activity participation because of motor coordination difficulties, sensory hypersensitivity, and motivational challenges. Screen-based gamified virtual reality (VR) sports have emerged as a promising alternative, with flow identified as a key psychological mechanism underlying their effects. However, alexithymia and atypical interoceptive processing in adolescents with ASD render conventional self-report flow scales inappropriate for this population. This study aimed to develop and validate a behaviorally anchored rating scale (BARS)-format proxy assessment tool, VR–Flow–BARS, that lets observers evaluate the flow-related behaviors of adolescents with ASD during VR sports participation.

**Methods:**

Eighteen preliminary items were developed from flow theory and the theoretical framework underlying the Flow State Scale-2. An eight-member expert panel of specialists in adapted physical education, ASD education, and VR sports evaluated the items against three criteria: content validity, observability, and appropriateness for ASD. The items were refined through cognitive interviews (*n* = 8) and pilot testing (*n* = 30). Exploratory factor analysis (EFA) using principal axis factoring with direct oblimin rotation was conducted on Sample 1 (*n* = 100), followed by confirmatory factor analysis (CFA) on Sample 2 (*n* = 200). Reliability was assessed using Cronbach’s α, composite reliability (CR), and the intraclass correlation coefficient (ICC) (2,1). Validity was examined using the average variance extracted (AVE), the Fornell–Larcker criterion, and the heterotrait–monotrait (HTMT) ratio.

**Results:**

The expert review yielded an item-level content validity index of 1.00 and a scale-level content validity index of 1.00 across all three criteria for the 15 retained items; the factor “sensorimotor flow expression” (with three items) was deleted for conceptual overlap and limited observability. EFA identified a five-factor structure accounting for 68.394% of the total variance. CFA demonstrated acceptable model fit (*χ*^2^/df = 2.000, comparative fit index = 0.969, Tucker–Lewis index = 0.959, root mean square error of approximatio*n* = 0.071), with standardized factor loadings ranging from 0.763 to 0.940. Internal consistency was high (Cronbach’s α = 0.864–0.946; CR = 0.868–0.946). Inter-rater reliability (ICC) ranged from 0.729 to 0.910 (indicating moderate to excellent reliability). AVE values ranged from 0.687 to 0.854, satisfying the convergent validity criterion of 0.50, and HTMT values for all factor pairs were below 0.90 at the point-estimate level; however, the 95% bootstrap confidence interval for the FR–CEP pair [0.851, 0.939] extended beyond 0.90, indicating borderline discriminant validity for this factor pair.

**Conclusion:**

To our knowledge, VR–Flow–BARS tool is the first BARS-format proxy assessment instrument developed to evaluate flow-related behaviors in adolescents with ASD during screen-based gamified VR sports. It demonstrates an acceptable factor structure, high internal consistency, adequate inter-rater reliability, and internal structural validity. This scale may serve as a practical tool for the real-time monitoring of immersive behaviors and individualized intervention design in VR-based physical activity programs for adolescents with ASD who have mild-to-moderate educational support needs and are able to engage in VR tasks with routine prompting. Future research should address external criterion-based validation, test–retest reliability, and applicability across a broader range of ASD severity levels.

## 1 Introduction

Adolescents with ASD face barriers to physical activity and exercise rehabilitation because of motor coordination difficulties, sensory hypersensitivity, and attention and motivation challenges; these constraints can harm their long-term health and social interaction skills (1). VR, exergames, and gamification-based interventions—including screen-based VR sports programs (2, 3), gamified mobile applications (4), and exergame-based physical therapy (6)—have recently drawn attention as alternatives for promoting physical activity and maximizing rehabilitation outcomes in this population.

Previous studies consistently show that gamification- and VR-based interventions can promote physical activity and produce positive changes in the motor, social, and emotional skills of adolescents with ASD (2–6). These interventions sustain interest and motivation while providing immediate feedback and appropriate challenges, thereby improving training quality. A recent VR exergaming study found that during active participation, energy expenditure rose significantly without a corresponding rise in perceived exertion (1), suggesting that VR may safely increase key exercise metrics—intensity and duration—by sustaining immersion and enjoyment.

Flow—an optimal state in which action and awareness are seamlessly integrated during deep task absorption (7)—has been proposed as a key mechanism underlying these effects. Clear goals, immediate feedback, and a challenge–skill balance are established conditions for eliciting flow in both sports (8, 9) and game-based contexts (10, 11). Flow has typically been measured using self-report instruments such as the Flow State Scale (12) and Flow Short Scale (13). However, three of the nine flow dimensions—“transformation of time,” “loss of self-consciousness,” and “autotelic experience”—are inherently subjective phenomenological states that cannot be directly operationalized as externally observable behaviors (29), a structural constraint for any proxy-based observational approach. Notably, the VR exergaming study cited above (1) proposed flow as the mechanism behind the intensity–exertion discrepancy but did not measure it directly— a limitation the authors explicitly acknowledged.

In this context, two compounding limitations affect self-report flow measurement. First, such scales are retrospective, requiring accurate recall of complex internal states after the activity ends—demanding even for typical respondents. Second, adolescents with ASD face additional constraints: many experience alexithymia—difficulty identifying and describing one’s own emotions (14)—and recent meta-analytic evidence shows altered interoceptive accuracy and sensibility relative to their typically developing peers (15). Therefore, self-report scales developed for the general population risk failing to capture or accurately reflect the actual experience of immersion in adolescents with ASD.

These limitations highlight the need for objective, observation-based assessments. Proxy assessment by caregivers or teachers has long been used to evaluate adaptive behavior, quality of life, and emotional characteristics in adolescents with limited communication or self-reporting ability (16, 17), and it offers a practical way to monitor real-time participation, attention, and task persistence. During VR sports, observable behaviors—gaze maintenance, speed of return to task after a stimulus, physical responses to feedback, and retry frequency after failure— can serve as behavioral proxies for flow. However, proxy assessment is vulnerable to rater subjectivity, halo effects, and inconsistent stringency, making abstract Likert-type response categories insufficient. Concrete, quantified behavioral criteria that anchor raters to shared, objective standards are therefore needed.

BARS address this need by anchoring each scale point to concrete, observable behavioral examples rather than vague impressions, thereby standardizing judgment across raters (18, 19). Its strength lies in its independence from any single domain, allowing its application across contexts as varied as healthcare, personnel selection, and education; it has been reported to improve inter-rater reliability wherever trained observers must judge complex behavior against a consistent standard (20–22). Assessing the VR sports participation of adolescents with ASD poses a similar structural challenge, differing only in that the target of judgment is not clinical performance or learning outcomes but the behavioral expression of an internal psychological state—namely, flow. Although flow is inherently internal, during VR sports it manifests through observable behaviors—attentional focus, goal-directed movement, and responses to feedback—making BARS particularly well suited to this context. Atypical behaviors common among adolescents with ASD— peripheral gaze, stereotypic behavior, prompt dependency, and delayed responses (31)—can be reinterpreted as atypical indicators of flow rather than measurement errors and written directly into the behavioral anchors, capturing patterns that general population flow scales fail to address (14, 15).

However, flow measurement in sports and VR still relies predominantly on self-report, and to our knowledge no BARS-based instrument has been developed to quantify ASD-specific immersion behavior during VR sports. Existing ASD observational tools likewise focus on diagnosis, symptom severity, or problem behavior screening rather than on positive immersive experiences, leaving clinicians without a standardized tool to monitor engagement and guide individualized intervention protocols in real time.

Against this background, the present study aimed to develop the VR–Flow–BARS proxy assessment scale—grounded in existing VR sports flow theory—to objectively assess the flow-related behaviors of adolescents with ASD during gamified VR sports and rehabilitation training, and to evaluate its reliability and validity. Specifically, ASD-specific behavioral indicators were derived from flow theory and existing scales, refined through expert content validity index (CVI) review, and subsequently evaluated using sequential EFA and CFA on independent samples, together with internal consistency, convergent validity, and discriminant validity. The resulting scale is intended to support real-time monitoring of immersive behavior and feedback responses in future VR-based rehabilitation programs for adolescents with ASD, thereby supporting safe, individualized intervention design.

## 2 Materials and methods

### 2.1 Design and procedure

**Figure 1:**
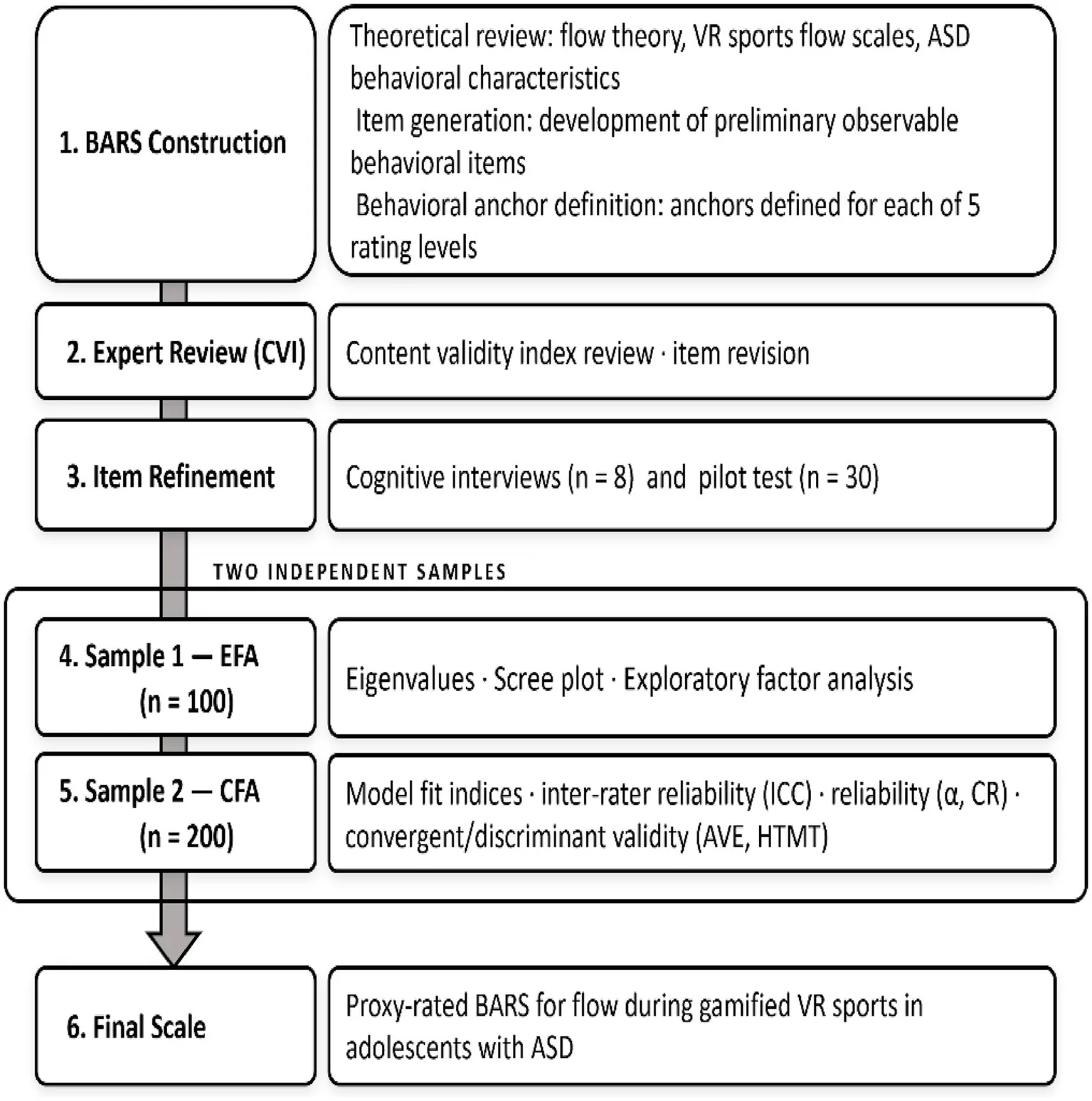
Development and validation of the VR–Flow–BARS proxy assessment scale for adolescents with ASD.

This study used a methodological design to develop and validate the VR–Flow–BARS proxy assessment scale. Scale development proceeded sequentially, as shown in Figure 1. Preliminary behavioral indicators and rating-level anchors reflecting the characteristics of adolescents with ASD were derived from a theoretical review of flow theory and existing VR sports flow scales. The items were subsequently revised following expert CVI review, and the comprehensibility of behavioral anchors was evaluated through small-scale cognitive interviews. Finally, the factor structure, reliability, and validity of the scale were examined through sequential EFA and CFA using two independent samples (Sample 1: *n* = 100; Sample 2: *n* = 200).

### 2.2 Participants

#### 2.2.1 Expert panel

An eight-member expert panel was recruited through snowball sampling to conduct the CVI review. All members had at least 10 years of experience in their respective fields. The panel comprised two specialists in adapted physical education and in measurement and evaluation (doctoral-level researchers focused primarily on psychometrics within adapted physical education), one specialist in adapted physical education (doctoral-level researcher), three specialists in ASD education (special education teachers), and two specialists in VR sports and exercise interventions (adapted physical education instructors). Panel members rated the relevance and clarity of each item on a 4-point scale and provided qualitative feedback on item revision or deletion.

#### 2.2.2 Adolescents with ASD

Participants were adolescents with ASD aged 13–18 years who were enrolled in special schools, special education classes within general schools, or disability sports and recreation facilities. The inclusion criteria were: (1) formal identification as having ASD through South Korea’s special education eligibility determination system; and (2) at least 15 weeks of participation in a school-or-community based screen-based VR/exergaming sports program, with the ability to perform the observed VR sports tasks under routine verbal or visual prompts and without continuous physical assistance.

As the Korean special education eligibility system identifies students primarily by disability category rather than by standardized ASD severity levels (30), no standardized support-level classification was available for the present study. Accordingly, eligibility was operationalized primarily in functional terms, using the participation criterion defined above. Functional eligibility was determined from educational records and from observations by the participant’s teacher or instructor in school- or community-based physical activity settings. The term “mild-to-moderate educational support needs” therefore refers to this functional participation criterion and should not be interpreted as a standardized clinical classification of ASD severity. No study-specific standardized diagnostic confirmation or autism symptom-severity measure (e.g., ADOS-2, CARS-2, or SRS-2) was administered, and these functional judgments were not independently double-rated. Adolescents requiring continuous physical assistance or intensive one-to-one support to participate in VR sports were excluded.

Here, “VR sports” refers to interactive, screen-based virtual sports activities in which participants engage with visual and auditory stimuli through whole-body movement on a standard display screen, as distinct from head-mounted display (HMD)-based fully immersive VR environments. Two independent samples were sequentially recruited according to the scale development procedure. Recruitment was conducted through cooperating special schools, special education classes within general schools, and disability sports and recreation facilities that offered screen-based VR/exergaming programs and were accessible to the research team. These institutions were not randomly selected from a national sampling frame; instead, a quota-based convenience sampling approach was used. The quota distribution—50% from special schools, 25% from special education classes within general schools, and 25% from disability sports and recreation facilities—was set *a priori* to reflect the relative availability of screen-based VR sports programs across setting types in South Korea at the time of data collection, when special schools had the highest rate of VR program adoption. Sample 1 (*n* = 100; 73 male, 27 female) was recruited first for EFA. Following item revision based on the EFA results, Sample 2 (*n* = 200; 148 male, 52 female) was recruited separately for CFA and validity assessment. No participant appeared in both samples.

#### 2.2.3 Proxy raters

Ratings were completed by special education teachers or adapted physical education instructors who had been responsible for each participant for at least 6 months and who directly observed the VR sports session. Each rating was based on a standardized 15-min observation window during the main activity phase of the session—excluding the initial familiarization or warm-up period—and was completed immediately afterward. The 15-min window was set with reference to prior autism observational coding and physical activity observation protocols that used brief, standardized observation periods in structured behavioral or activity contexts (23, 24). The rater structure was designed to reflect field conditions in special education and adapted physical activity settings, where a single teacher or instructor typically observes and evaluates several adolescents in the same session. Accordingly, each proxy rater evaluated five participants rather than one, to enhance the ecological validity and practical applicability of VR–Flow–BARS as a real-world monitoring tool. The rater pool comprised 20 raters in Sample 1 (predominantly special education teachers) and 40 raters in Sample 2.

Rater training consisted of a one-time group session conducted by video conferencing, followed by detailed written guidelines distributed by email. The training and guidelines covered the study purpose, rating procedures, interpretation of the behavioral description statements for each item, and key considerations during observation. An ongoing communication channel was also maintained throughout data collection, so raters could contact the research team at any time with questions about item interpretation or rating procedures.

Because multiple participants were rated by the same proxy rater, rater-specific leniency or severity effects could not be completely ruled out. To address this issue, the number of participants evaluated by each proxy rater was fixed at five, and potential rater-level dependency was additionally examined statistically as described in the Data Analysis section. In addition, approximately 30% of the participants in Sample 2 were independently double-rated by two proxy raters to assess inter-rater reliability under field-based conditions.

### 2.3 Ethical considerations

This study was approved by the Institutional Review Board (IRB) of Korea National Sport University, Seoul, Republic of Korea [KNSU IRB 20221209–095; December 9, 2022]. Written informed consent was obtained from the legal guardians of all participants after they received a full explanation of the study purpose, procedures, potential risks and benefits, and their right to withdraw at any time without penalty. Because many participants had limited verbal communication, obtaining direct assent from them was not feasible in most cases; therefore, written informed consent was obtained from legal guardians. When possible, participants’ verbal or nonverbal assent was also sought. For those with limited communication, participation continued only if no signs of distress, refusal, or withdrawal were observed. Written informed consent was also obtained from all expert panel members who participated in the content validity review and from all proxy raters who conducted the behavioral assessments. All data were anonymized and used solely for research purposes.

### 2.4 Scale development

#### 2.4.1 Item development

Preliminary items for the VR–Flow–BARS tool were derived from Csikszentmihalyi’s nine-dimensional flow theory (7) and from the frameworks underpinning the Flow State Scale-2 (FSS-2) and Dispositional Flow State Scale-2 (DFS-2), developed by Jackson and Eklund (25) to operationalize the nine flow dimensions for physical activity contexts. Of these, six were selected for their potential to be expressed as externally observable behaviors during gamified VR sports participation among adolescents with ASD. Three dimensions—transformation of time, loss of self-consciousness, and autotelic experience—were excluded on theoretical grounds. As Nakamura and Csikszentmihalyi (29) noted, these dimensions are qualitatively distinct from the remaining six because they are phenomenological states experienced during the flow episode rather than behavioral dispositions or responses. Therefore, they are inherently difficult for external observers to infer from behavioral indicators. This constraint is compounded for adolescents with ASD, whose characteristics— including alexithymia (14) and atypical interoceptive processing (15)—make it especially challenging to operationalize the behavioral expression of these deeply internal states. Table 1 summarizes the nine flow dimensions proposed by Csikszentmihalyi (7) and operationalized in the FSS-2/DFS-2 (25), along with the inclusion or exclusion decision and rationale applied in the present study.

**Table 1:** Mapping of flow theory dimensions to VR–flow–BARS development decisions.

Flow dimension (7, 25)	Decision	Behavioral observability rationale	VR-flow-BARS factor
Challenge–skill balance	Included	Observable through behavioral persistence, effort regulation, and engagement following difficulty changes	Challenge Engagement and Persistence (CEP)
Clear goals	Included	Observable through goal-directed movement, target orientation, and task-aligned actions	Task Understanding and Goal Orientation (TG)
Unambiguous feedback	Included	Observable through physical and gaze-based responses to VR visual and auditory feedback signals	VR Feedback Responsiveness (FR)
Concentration on the task	Included	Observable through sustained gaze orientation and reduced off-task behavior	Motor Concentration (MC)
Merging of action and awareness	Included (combined)	Observable through spontaneous, fluid movement initiation without external prompting	Autonomous Motor Engagement (AME)
Sense of control	Included (combined)	Observable through independent motor engagement and autonomous participation	Autonomous Motor Engagement (AME)
Loss of self-consciousness	Excluded	Inherently subjective phenomenological state; not reliably inferrable from external behavior; particularly inaccessible in ASD due to alexithymia (14) and atypical interoceptive processing (15)	—
Transformation of time	Excluded	Requires introspective awareness of temporal perception; cannot be operationalized as an external behavioral indicator (29)	—
Autotelic experience	Excluded	Inherently subjective and self-referential; not directly inferrable from external behavior; particularly inaccessible in ASD due to alexithymia (14) and atypical interoceptive processing (15)	—

The merging of action and awareness and sense of control were combined into a single factor (AME) because both manifest as behavioral expressions of spontaneous, self-initiated motor engagement in the VR sports context. An additional ASD-specific candidate factor, sensorimotor flow expression, was developed to capture timing congruence and sensorimotor responsiveness unique to this population; however, it received I-CVI = 0.00 across all three expert review criteria and was deleted (see Results: Content Validity).

The initial six factors were: (1) motor concentration, (2) task understanding and goal orientation, (3) VR feedback responsiveness, (4) challenge engagement and persistence, (5) autonomous motor engagement, and (6) sensorimotor flow expression. Each factor comprised three items, yielding a total of 18 preliminary items.

Following the BARS format, each item was structured on a five-point scale ranging from 1 (behavior not observed) to 5 (behavior clearly and consistently observed), accompanied by behavioral description statements for each rating level. These statements were developed to treat atypical characteristics commonly observed in adolescents with ASD (e.g., peripheral gaze processing, stereotypic behavior, prompt dependency, and delayed responses) as behavioral manifestations tied to different levels of flow, thereby integrating ASD-specific patterns that general-population flow scales have missed.

#### 2.4.2 Expert content validity review

The eight-member expert panel evaluated content validity for all 18 preliminary items. Each item was rated on three criteria—(1) content validity, (2) observability, and (3) appropriateness for ASD—each one on a four-point scale (1 = not at all appropriate, 4 = highly appropriate). Applying these three criteria was intended to ensure not only adequate content validity but also the observability and population specificity a proxy-based BARS instrument requires.

Items with an I-CVI below 0.78 were revised or removed (26). The three items making up Factor 6 (sensorimotor flow expression) were deleted; the rationale is discussed in detail in the Results. The final scale comprised 15 items across five factors: (1) motor concentration, (2) task understanding and goal orientation, (3) VR feedback responsiveness, (4) challenge engagement and persistence, and (5) autonomous motor engagement. The scale-level content validity index (S-CVI/Ave) was also calculated.

#### 2.4.3 Cognitive interviews

Cognitive interviews were conducted with eight special education teachers and adapted physical education instructors to assess whether the behavioral description statements in the revised 15-item scale were understood as intended in real VR sports observation settings. No items required structural modification following the interviews; only minor wording revisions were made to align terminology with the language commonly used in the field.

#### 2.4.4 Pilot test

A pilot test was conducted with 30 adolescents with ASD (independent of Samples 1 and 2) using the 15 items refined through cognitive interviews. It examined the basic statistical properties of each item: descriptive statistics (mean, standard deviation (SD), skewness, and kurtosis), rates of missing data, corrected item-total correlations (criterion: ≥0.30), and inter-item correlations. No items showed serious statistical problems, and the five-factor, 15-item structure was retained as the final scale in the main study.

### 2.5 Data analysis

All data were analyzed in SPSS and AMOS (version 31.0, IBM Corp., Armonk, NY, USA). The analyses proceeded in the following sequence. First, descriptive statistics and normality screening were performed: the mean, SD, skewness, and kurtosis were calculated for all items, and items with absolute skewness values above 2.0 or absolute kurtosis values above 7.0 were flagged as potentially violating the normality assumptions (35). Second, item analysis examined corrected item-total correlations (deletion criterion <0.30) and the change in Cronbach’s α on item deletion, to assess basic statistical adequacy at the item level. Third, EFA was conducted on Sample 1 (*n* = 100). The number of factors was determined by integrating the theoretical structure of flow theory, the five-factor structure established through expert content validity review, and the interpretability of the pattern matrix. Factors were extracted in SPSS using principal axis factoring (PAF) with direct oblimin oblique rotation (δ = 0). Oblique rotation was preferred over orthogonal rotation given the anticipated intercorrelations among the factors. A factor loading of 0.40 or above was used as the criterion for meaningful factor loadings. Fourth, CFA was conducted on Sample 2 (*n* = 200) in AMOS. Maximum likelihood (ML) estimation was used with the Bollen–Stine bootstrap correction to account for potential violations of multivariate normality. Model fit was evaluated using the *χ*^2^/df ratio, comparative fit index (CFI), Tucker–Lewis index (TLI), and root mean square error of approximation (RMSEA). Acceptable fit criteria were set as *χ*^2^/df < 3.0, CFI ≥ 0.90, TLI ≥ 0.90, and RMSEA≤0.08. The CFA sample size of 200 was based on established guidelines for structural equation modeling. Wolf et al. (36) showed through simulation that models with high standardized factor loadings (≥ 0.70), three indicators per factor, and five factors require approximately 130–200 participants for adequate statistical power and solution stability. Because all standardized factor loadings in the present model ranged from 0.763 to 0.940, *n* = 200 meets this criterion and yields a participant-to-indicator ratio of approximately 13:1, exceeding the commonly recommended minimum of 10:1 (37). The Bollen-Stine bootstrap correction was also applied to account for potential violations of multivariate normality, providing a further safeguard against sample-size sensitivity.

Fifth, reliability was assessed. Internal consistency was measured with Cronbach’s α, and composite reliability (CR) was calculated from the standardized factor loadings of the CFA model. Criteria of α ≥ 0.70 and CR ≥ 0.70 were applied. Sixth, convergent and discriminant validity were assessed. Convergent validity was evaluated with the average variance extracted (AVE; criterion≥0.50). Discriminant validity was evaluated with both the Fornell–Larcker criterion (the square root of each factor’s AVE should exceed its correlations with all other factors) and the heterotrait–monotrait (HTMT) ratio (criterion: <0.90). Seventh, inter-rater reliability was calculated as the ICC (2, 1); two-way random-effects model, absolute agreement, and single-measures using the SPSS reliability analysis procedure. Reliability was interpreted according to Koo and Li’s (27) benchmarks (<0.50 poor, 0.50–0.75 moderate, 0.75–0.90 good, ≥0.90 excellent). Eighth, potential rater-level dependency from several participants being evaluated by the same proxy rater was examined using one-way random-effects models, with proxy rater as the grouping variable (38). Rater-level ICCs were estimated for the five factor scores in both Sample 1 and Sample 2. Because each proxy rater evaluated five participants, design effects were calculated as 1 + (m − 1)ICC, where *m* = 5 (39). Negative ICC estimates, which can occur when the between-rater mean square is smaller than the within-rater mean square, were taken to indicate essentially no rater-level clustering and were treated as zero when calculating design effects.

## 3 Results

### 3.1 Content validity

The eight-member expert panel evaluated the 18 preliminary items on three criteria: content validity, observability, and appropriateness for ASD. All 15 items retained in the final five-factor structure received an I-CVI of 1.00 across the three criteria, and the S-CVI/Ave was 1.000 for each, exceeding the 0.90 threshold proposed by Polit, Beck, and Owen (26).

The three items constituting Factor 6 (sensorimotor flow expression) received I-CVI values of 0.00 across all three criteria, well below the deletion threshold of 0.78. The experts noted that these items risked conceptual overlap with motor concentration and VR feedback responsiveness in sensorimotor timing and response latency, and that rating the qualitative characteristics of sensorimotor expression in real time would require considerable professional interpretation. This was judged incompatible with the core BARS principle of standardizing inter-rater interpretation, therefore the factor was deleted in its entirety.

After item wording was revised in response to the panel’s qualitative feedback, cognitive interviews were conducted with eight teachers and instructors, after which minor terminology revisions were made, as described above.

### 3.2 Pilot test results

The pilot test with 30 adolescents with ASD (independent of Samples 1 and 2) used the finalized VR–Flow–BARS, comprising 15 items across five factors. Item-level statistical adequacy was examined as follows.

Item means ranged from 3.93 to 4.40 and SDs from 0.67 to 1.05. Skewness values ranged from −1.052 to −0.259, and kurtosis from −1.457 to 0.925. These values fell within the acceptable thresholds (absolute skewness <2.0, absolute kurtosis <7.0), indicating no serious violation of the normality assumptions. No item had missing data. Corrected item-total correlations exceeded the 0.30 criterion for all items, ranging from 0.317 to 0.781. The five-factor, 15-item structure was retained for the main study.

### 3.3 Participant characteristics

Participant characteristics for Samples 1(EFA) and 2 (CFA) are shown in Table 2. Sample 1 comprised 100 adolescents with ASD (73 male, 73.0%; 27 female, 27.0%), with a mean age of 15.7 years (SD = 0.7). By recruitment setting, 50 participants (50.0%) were from special schools, 25 (25.0%) from special education classes within general schools, and 25 (25.0%) from disability sports and recreation facilities. Twenty proxy raters each evaluated five participants, with a mean rater experience of 13.43 years.

**Table 2:** Participant characteristics.

Characteristic	Sample 1, EFA (*n* = 100)	Sample 2, CFA (*n* = 200)
Adolescents with ASD, *n*	100	200
Male, *n* (%)	73 (73.0)	148 (74.0)
Female, *n* (%)	27 (27.0)	52 (26.0)
Age, mean ± SD	15.7 ± 0.7 (13–18)	16.1 ± 1.2 (13–18)
Special school, *n* (%)	50 (50.0)	100 (50.0)
Special class, *n* (%)	25 (25.0)	50 (25.0)
Disability sports facility, *n* (%)	25 (25.0)	50 (25.0)
Proxy raters, *n*	20	40
Mean rater experience, years	13.43	12.68
Participants per rater	5	5
Double-rated participants, *n* (%)	–	60 (30%)

Sample 2 comprised 200 adolescents with ASD (148 male, 74.0%; 52 female, 26.0%), with a mean age of 16.1 years (SD = 1.2). The distribution by recruitment setting matched Sample 1: with 100 participants (50.0%) from special schools, 50 (25.0%) from special education classes within general schools, and 50 (25.0%) from disability sports and recreation facilities. Forty proxy raters each evaluated five participants, with a mean rater experience of 12.68 years. To assess inter-rater reliability, 60 participants in Sample 2 (30.0%) were independently double-rated. The two samples had highly similar distributions of sex, recruitment setting, and participants-per-rater ratio.

### 3.4 EFA

EFA was conducted on Sample 1 (*n* = 100) using PAF with direct oblimin oblique rotation. Before factor extraction, the Kaiser–Meyer–Olkin (KMO) measure of sampling adequacy and Bartlett’s test of sphericity were examined. The KMO value was 0.708 and Bartlett’s test was significant, *χ*^2^(105) = 358.323, *p* < 0.001, indicating that the data were suitable for factor analysis.

The number of factors to retain was determined by integrating the initial eigenvalues, the theoretical structure of flow theory, the five-factor structure from the expert content validity review, and the interpretability of the pattern matrix.

The first five eigenvalues exceeded 1.0 (6.186, 1.769, 1.511, 1.231, and 1.019) After PAF extraction, the five-factor solution accounted for 68.394% of the total variance. Given these results and the theoretical interpretability of the factor structure, a five-factor solution was retained.

The pattern matrix is presented in Table 3. All 15 items demonstrated primary factor loadings of 0.40 or above on their intended factors, while cross-loadings on other factors remained below 0.40. These results indicate that each item loaded onto a single factor. Factor 1 comprised TG1, TG2, and TG3, with loadings ranging from 0.693 to 0.839, and was labeled Task Understanding and Goal Orientation. Factor 2 comprised MC1, MC2, and MC3, with loadings ranging from 0.542 to 0.874, and was labeled Motor Concentration. Factor 3 comprised AME1, AME2, and AME3, with loadings ranging from 0.766 to 0.853, and was labeled Autonomous Motor Engagement. Factor 4 comprised CEP1, CEP2, and CEP3, with loadings ranging from 0.533 to 0.921, and was labeled Challenge Engagement and Persistence. Factor 5 comprised FR1, FR2, and FR3, with loadings ranging from 0.619 to 0.995, and was labeled VR Feedback Responsiveness.

**Table 3:** Pattern matrix from exploratory factor analysis (sample 1, *n* = 100).

Item	Factor 1 (TG)	Factor 2 (MC)	Factor 3 (AME)	Factor 4 (CEP)	Factor 5 (FR)
TG1	**0.839**	0.007	0.110	0.003	0.036
TG2	**0.822**	0.033	0.048	0.062	0.021
TG3	**0.693**	0.054	0.030	0.087	0.083
MC1	0.165	**0.874**	0.109	0.010	0.061
MC2	0.067	**0.719**	0.015	0.030	0.050
MC3	0.131	**0.542**	0.063	0.039	0.002
AME1	0.092	0.068	**0.853**	0.061	0.062
AME2	0.049	0.105	**0.825**	0.113	0.172
AME3	0.060	0.000	**0.766**	0.125	0.042
CEP1	0.073	0.017	0.068	**0.921**	0.020
CEP2	0.072	0.032	0.051	**0.786**	0.016
CEP3	0.014	0.097	0.135	**0.533**	0.128
FR1	0.074	0.037	0.005	0.042	**0.995**
FR2	0.181	0.075	0.028	0.145	**0.662**
FR3	0.169	0.005	0.053	0.086	**0.619**

PAF, principal axis factoring; direct oblimin rotation (δ = 0). Loadings ≥0.40 are shown in bold. TG, task understanding and goal orientation; MC, motor concentration; AME, autonomous motor engagement; CEP, challenge engagement and persistence; FR, VR feedback responsiveness. Bold values indicate factor loadings of 0.40 or above, which were used as the criterion for meaningful factor loadings in the present study. Items with primary loadings ≥ 0.40 on their intended factor are shown in bold to distinguish them from cross-loadings on other factors.

### 3.5 CFA

CFA was conducted on Sample 2 (*n* = 200) to verify the five-factor, 15-item structure from the EFA. Model fit indices were as follows: *χ*^2^(80) = 160.007, *χ*^2^/df = 2.000, CFI = 0.969, TLI = 0.959, and RMSEA = 0.071(90% CI: 0.055–0.087). All indices met the predetermined criteria (*χ*^2^/df < 3.0, CFI ≥ 0.90, TLI ≥ 0.90, RMSEA≤0.08). The Bollen–Stine bootstrap result was nonsignificant (*p* = 0.220), indicating that the model fit remained acceptable after correction for potential multivariate non-normality.

The standardized factor loadings are listed in Table 4. All 15 items showed high standardized loadings on their respective factors, ranging from 0.763 to 0.940, confirming strong relationships between the items and their corresponding latent factors. Factor-level loadings were as follows: task understanding and goal orientation (TG1 = 0.940, TG2 = 0.925, TG3 = 0.907), motor concentration (MC1 = 0.838, MC2 = 0.835, MC3 = 0.814), autonomous motor engagement (AME1 = 0.876, AME2 = 0.861, AME3 = 0.763), challenge engagement and persistence (CEP1 = 0.933, CEP2 = 0.883, CEP3 = 0.871), and VR feedback responsiveness (FR1 = 0.927, FR2 = 0.848, FR3 = 0.796). These results support the five-factor structure of the VR–Flow–BARS tool for assessing flow-related behaviors in adolescents with ASD during gamified VR sports.

**Table 4:** Standardized factor loadings from confirmatory factor analysis (sample 2, *n* = 200).

Factor	Items	*β* range
Task understanding and goal orientation	TG1, TG2, TG3	0.907–0.940
Motor concentration	MC1, MC2, MC3	0.814–0.838
Autonomous motor engagement	AME1, AME2, AME3	0.763–0.876
Challenge engagement and persistence	CEP1, CEP2, CEP3	0.871–0.933
VR Feedback responsiveness	FR1, FR2, FR3	0.796–0.927

*β*, standardized factor loading (range across three items per factor); all loadings were significant at *p* < 0.001. Model fit (Sample 2, *n* = 200): *χ*^2^(80) = 160.007, *χ*^2^/df = 2.000, CFI = 0.969, TLI = 0.959, RMSEA = 0.071(90% CI: 0.055–0.087), Bollen–Stine bootstrap *p* = 0.220.

### 3.6 Reliability

Internal consistency, CR, and inter-rater reliability of VR–Flow–BARS were assessed; the results are presented in Table 5.

**Table 5:** Reliability indices of the VR–flow–BARS tool (sample 2, *n* = 200).

Factor	*α*	CR	ICC (2,1)	95% CI
Task understanding and goal orientation	0.870	0.946	0.795	0.679–0.872
Motor concentration	0.924	0.868	0.910	0.854–0.945
Autonomous motor engagement	0.864	0.873	0.729	0.674–0.870
Challenge engagement and persistence	0.892	0.924	0.744	0.605–0.839
VR feedback responsiveness	0.946	0.893	0.874	0.797–0.923

α, Cronbach’s alpha; CR, composite reliability; ICC (2,1) = intraclass correlation coefficient (two-way random effects model, absolute agreement, single measures); CI, confidence interval. ICC benchmarks from Koo and Li’s study (27): <0.50 poor, 0.50–0.75 moderate, 0.75–0.90 good, ≥0.90 excellent.

Cronbach’s α ranged from 0.864 (AME) to 0.946 (FR), exceeding the 0.70 criterion for every factor. CR ranged from 0.868 (MC) to 0.946 (TG), also above 0.70 for all factors. These results support high internal consistency across all five factors of the VR–Flow–BARS tool.

For inter-rater reliability, two independent raters double-rated 60 participants (30%) in Sample 2, and reliability was estimated with ICC (2,1). Factor-level ICC (2,1) values ranged from 0.729 (AME) to 0.910 (MC). By Koo and Li’s (27) benchmarks, MC (ICC = 0.910) was classified as excellent, TG (ICC = 0.795) and FR (ICC = 0.874) were good, and AME (ICC = 0.729) and CEP (ICC = 0.744) were moderate.

### 3.7 Rater-level dependency

To examine potential dependency from multiple participants being evaluated by the same proxy rater, rater-level ICCs were estimated using one-way random-effects models, with proxy rater as the grouping variable. In Sample 1, rater-level ICC estimates ranged from −0.008 to 0.040; the negative estimates were taken to indicate essentially no rater-level clustering. The corresponding design effects ranged from approximately 1.000 to 1.162, and no statistically significant between-rater differences emerged across the five factors (ps = 0.272–0.515). In Sample 2, rater-level ICCs ranged from 0.020 to 0.067, with design effects ranging from 1.082 to 1.269. Again, no statistically significant between-rater differences emerged across the five factors (ps = 0.095–0.328). Overall, these findings indicate that rater-level dependency was modest in both the EFA and CFA samples (Table 6).

**Table 6:** Rater-level dependency of VR–flow–BARS factor scores.

Factor	MS between	MS within	*F*	*p*	Rater-level ICC	Design effect
Sample 1 (*n* = 100; 20 proxy raters × 5 participants)						
TG	0.574	0.598	0.960	0.515	−0.008	≈1.000
MC	0.574	0.474	1.209	0.272	0.040	1.162
AME	0.396	0.398	0.995	0.475	−0.001	≈1.000
CEP	0.496	0.467	1.062	0.405	0.012	1.049
FR	0.496	0.422	1.174	0.301	0.034	1.136
Sample 2 (*n* = 200; 40 proxy raters × 5 participants)						
TG	0.500	0.387	1.294	0.137	0.055	1.221
MC	0.650	0.478	1.362	0.095	0.067	1.269
AME	0.502	0.416	1.207	0.210	0.040	1.159
CEP	0.598	0.488	1.225	0.193	0.043	1.173
FR	0.498	0.451	1.104	0.328	0.020	1.082

ICC, intraclass correlation coefficient estimated from a one-way random-effects model with proxy rater as the grouping variable; design effect = 1 + (*m* − 1)ICC, where *m* = 5 participants per proxy rater. Negative ICC estimates were interpreted as indicating essentially no rater-level clustering and were treated as zero when calculating the design effect.

### 3.8 Convergent and discriminant validity

Convergent validity was assessed using the AVE, which ranged from 0.687 (MC) to 0.854 (TG), meeting the 0.50 criterion for all factors.

Discriminant validity was evaluated using both the Fornell–Larcker criterion and the HTMT ratio. The FR–CEP inter-factor correlation (0.910) exceeded the square roots of the AVE for both FR (0.858) and CEP (0.896), indicating that the Fornell–Larcker criterion was not satisfied for this pair. The HTMT point estimate was 0.899, and bootstrap analysis based on 5,000 resamples yielded a 95% confidence interval of [0.851, 0.939], with the upper bound exceeding the 0.90 threshold (28). Thus, discriminant validity between FR and CEP was considered borderline rather than conclusively established. HTMT values for the remaining nine factor pairs were all below 0.85 (Table 7).

**Table 7:** Convergent and discriminant validity of the VR–flow–BARS tool (sample 2, *n* = 200).

Factor	AVE	TG	MC	AME	CEP	FR
Fornell–Larcker criterion						
TG	0.854	0.924				
MC	0.687	0.505	0.829			
AME	0.697	0.417	0.613	0.835		
CEP	0.803	0.430	0.641	0.649	0.896	
FR	0.737	0.414	0.724	0.599	0.910*	0.858
HTMT						
TG	—	—				
MC	—	0.552	—			
AME	—	0.462	0.715	—		
CEP	—	0.474	0.740	0.749	—	
FR	—	0.457	0.841	0.694	0.899	—

AVE, average variance extracted. Diagonal (bold), square root of AVE. Lower triangle (Fornell–Larcker), inter-factor correlations. Lower triangle (HTMT), Heterotrait–Monotrait ratio of correlations. TG, task understanding and goal orientation; MC, motor concentration; AME, autonomous motor engagement; CEP, challenge engagement and persistence; FR, VR feedback responsiveness. *The FR–CEP correlation (0.910) exceeds the square roots of the AVE for both FR (0.858) and CEP (0.896), so the Fornell–Larcker criterion is not satisfied for this pair. The HTMT point estimate (0.899) is marginally below the 0.90 threshold; however, the 95% bootstrap confidence interval [0.851, 0.939] extends beyond 0.90 (28), so discriminant validity between these two factors should be regarded as borderline.

To further examine possible conceptual redundancy between FR and CEP, and to determine whether a more parsimonious structure would better represent the data, an alternative four-factor model merging FR and CEP into a single factor was evaluated. Its fit indices did not meet the acceptable thresholds: *χ*^2^/df = 4.436, CFI = 0.885, TLI = 0.858, and RMSEA = 0.131 (90% CI: 0.118–0.145). Compared with the hypothesized five-factor model (*χ*^2^/df = 2.000, CFI = 0.969, TLI = 0.959, RMSEA = 0.071), the collapsed model showed substantially poorer fit. These findings provide complementary empirical support for retaining FR and CEP as separate factors in the five-factor model despite their substantial overlap.

## 4 Discussion

This study developed and psychometrically validated a behaviorally anchored proxy rating scale (VR–Flow–BARS) to assess the flow-related behaviors of adolescents with ASD during screen-based gamified VR sports participation. Following a development process of expert content validity review, cognitive interviews, pilot testing, and sequential EFA and CFA on two independent samples, a final scale of 15 items across five factors was established. Overall, the VR–Flow–BARS tool demonstrated an acceptable factor structure, high internal consistency, adequate inter-rater reliability, and acceptable convergent validity, supporting its potential as a behavioral proxy measure of flow in adolescents with ASD for whom self-report is not feasible.

### 4.1 Factor structure and content validity

The five-factor structure was grounded in the dimensions of Csikszentmihalyi’s flow theory (7) and the FSS-2/DFS-2 framework of Jackson and Eklund (25), with factors selected for their potential to manifest as externally observable behaviors in gamified VR sports. The five retained factors—task understanding and goal orientation, motor concentration, autonomous motor engagement, challenge engagement and persistence, and VR feedback responsiveness—represent the behavioral expressions of core flow conditions, including clear goal orientation, focused attention, immediate feedback response, challenge–skill balance, and self-initiated persistence, thereby lending theoretical coherence to the factor structure.

The eight-member expert panel assigned I-CVI = 1.00 and S-CVI/Ave = 1.00 across all three criteria for the 15 retained items—high content validity relative to prior BARS development studies (20, 21). This indicates that the field practitioners saw the items as clear, observable, and appropriate for the ASD population. By contrast, the sixth candidate factor, sensorimotor flow expression, received an I-CVI = 0.00 across all three criteria. The experts indicated that the constructs underlying this factor—sensorimotor timing congruence and response latency—risked conceptual overlap with motor concentration and VR feedback responsiveness, and that assessing them in real time in the field would demand a level of professional interpretation inconsistent with the core BARS principle of standardized inter-rater interpretation. A more fundamental concern is that stereotypic motor behavior, a core diagnostic criterion of ASD (31), carries an inherent interpretive ambiguity: the same behavioral expression may signify heightened engagement and arousal or anxiety-driven avoidance, making it extremely difficult for raters to apply consistent criteria. Thus, even when sensorimotor expressions are externally observable, their meaning remains heavily dependent on subjective rater judgment, failing to satisfy the foundational BARS requirement for concrete, unambiguous behavioral anchors (18, 19). Therefore, the deletion of this factor was a principled psychometric decision that preserved the methodological integrity of the BARS approach.

The five retained factors represent a theoretically grounded reconceptualization of the core flow conditions as externally observable behavioral dimensions, with each factor mapping onto a specific element of Csikszentmihalyi’s flow theory (7, 29). Task understanding and goal orientation correspond to the condition of clear goals; motor concentration corresponds to concentration on the task at hand; autonomous motor engagement corresponds to action–awareness merging and the sense of control; challenge engagement and persistence corresponds to challenge–skill balance; and VR feedback responsiveness corresponds to unambiguous feedback. The three excluded dimensions—transformation of time, loss of self-consciousness, and autotelic experience—are, as Nakamura and Csikszentmihalyi (29) described, phenomenological outcomes experienced internally during the flow episode and cannot be reliably inferred from behavioral observations. This rationale is reinforced by the characteristics of adolescents with ASD, for whom alexithymia (14) and atypical interoceptive processing (15) make the behavioral expression of such deeply internal states especially difficult to operationalize. Accordingly, the five-factor structure of the VR–Flow–BARS tool reflects a deliberate design choice balancing theoretical fidelity with observational feasibility in a proxy-based assessment context.

### 4.2 Psychometric properties

CFA confirmed the five-factor structure, with fit indices (*χ*^2^/df = 2.000, CFI = 0.969, TLI = 0.959, and RMSEA = 0.071) all meeting the predetermined criteria. Standardized factor loadings ranged from 0.763 to 0.940, confirming strong relationships between the items and their factors. For reliability, Cronbach’s α ranged from 0.864 to 0.946 and CR ranged from 0.868 to 0.946, exceeding 0.70 for every factor. AVE values ranged from 0.687 to 0.854, meeting the 0.50 criterion for all factors.

Although the upper Cronbach’s α and CR were high—particularly for three-item factors—high internal consistency does not necessarily imply optimal measurement quality and can sometimes reflect item redundancy (42, 43). In this scale, however, the three items within each factor were developed to represent conceptually distinct, observable manifestations of the same underlying behavioral domain rather than paraphrased versions of a single behavior. This distinction was explicitly considered during expert content validity review and cognitive interviews. Thus, the high reliability coefficients are interpreted as reflecting strong within-factor coherence, although the possibility of partial item redundancy should still be examined in future replication studies.

### 4.3 Discriminant validity and the FR–CEP inter-factor correlation

One notable finding was the high inter-factor correlation (0.910) between VR feedback responsiveness (FR) and challenge engagement and persistence (CEP). The Fornell–Larcker criterion was not satisfied for this pair, because the correlation exceeded the square root of the AVE for both FR (0.858) and CEP (0.896). Although the HTMT point estimate (0.899) was marginally below the 0.90 threshold (28), bootstrap analysis yielded a 95% confidence interval of [0.851, 0.939], with the upper bound exceeding 0.90. Thus, discriminant validity between FR and CEP should be regarded as borderline rather than conclusively established (28). Nevertheless, the alternative four-factor model merging FR and CEP showed substantially poorer fit (*χ*^2^/df = 4.436, CFI = 0.885, TLI = 0.858, RMSEA = 0.131) than the hypothesized five-factor model (*χ*^2^/df = 2.000, CFI = 0.969, TLI = 0.959, RMSEA = 0.071). This provides complementary empirical support for retaining FR and CEP as separate factors despite their substantial overlap.

From a theoretical perspective, the high inter-factor correlation between FR and CEP may reflect a convergence of ASD-specific sensory processing characteristics and the structural properties of the VR environment. Individuals with ASD have been shown to rely atypically on unimodal visual feedback rather than on internal proprioceptive feedback during motor performance, owing to deficits in multisensory processing (32). In a VR environment that provides continuous, structured visual feedback, this external signal mediates both responses to task feedback (FR) and persistence through challenges (CEP). Flow theory specifies that unambiguous feedback is a necessary precondition for challenge–skill balance (7, 29), and for adolescents with ASD, whose interoceptive awareness is atypical (15), the external visual feedback may serve as a shared regulatory mechanism linking these two behavioral dimensions. One interpretation is that this pattern reflects a context-specific relationship between feedback responsiveness and challenge persistence in visually structured VR environments; however, it remains tentative and was not directly tested in this study. Future research should examine whether this association is modulated by ASD severity, VR experience, or feedback delivery format, and should further refine the behavioral description statements for FR and CEP. Replication in independent samples will also be necessary to determine whether the distinction between these two dimensions remains stable across populations and VR contexts.

### 4.4 Inter-rater reliability

Factor-level ICC (2,1) values ranged from 0.729 (AME) to 0.910 (MC). Motor concentration was excellent; task understanding and goal orientation and VR feedback responsiveness were good; and autonomous motor engagement and challenge engagement and persistence were moderate. For AME, prompt dependency is a common concern among individuals with ASD, often making truly independent initiation difficult to observe (33). During VR sports participation, the visual onset of the screen can act as a natural environmental cue that triggers movement, making it structurally ambiguous for raters to determine whether a student’s initiation reflects spontaneous engagement or a conditioned response to an environmental prompt. For CEP, when instructors adjust task difficulty in real time in response to student distress, rater judgments about whether persistence reflects genuine challenge engagement may become context-dependent, potentially lowering inter-rater agreement (20). Reliance on video-conference group training and written guidelines, without in-person calibration sessions, may also have contributed to variability in rater interpretation. Future research should therefore incorporate in-person calibration sessions using video-based behavioral examples, with specific attention to distinguishing spontaneous from environment-prompted initiation in AME, and genuine challenge persistence from task-accommodation-driven continuation in CEP.

Beyond inter-rater agreement, potential dependency from multiple participants being evaluated by the same proxy rater was also examined (38). Rater-level ICCs ranged from approximately zero to 0.040 in Sample 1 and 0.020 to 0.067 in Sample 2, and no statistically significant between-rater differences emerged across the five factors in either sample. These findings suggest that systematic rater-level clustering was modest under this rating. This analysis complements rather than replaces the inter-rater ICC(2,1): the former assesses dependency among different participants evaluated by the same proxy rater, whereas the latter assesses agreement between independent proxy raters evaluating the same participant (40). Nevertheless, residual rater-specific leniency or severity effects cannot be completely excluded (38, 41).

### 4.5 Theoretical contributions

A key theoretical contribution of VR–Flow–BARS tool is that it reframes flow from a purely self-reported phenomenological state into a set of observable, context-sensitive behavioral indicators applicable to adolescents with ASD during VR sports participation. The flow measurement literature suggests that overreliance on subjective self-report limits the refinement of the flow construct and its discriminant validity relative to adjacent constructs such as motivation or optimal task performance (34). Despite the recognized importance of capturing the behavioral expression of flow in sports contexts (9), existing flow scales have predominantly assumed that individuals can retrospectively identify and report internal states such as absorption, control, and enjoyment. By contrast, the VR–Flow–BARS tool operationalizes flow through externally observable patterns of task focus, goal-directed movement, feedback response, persistence through challenges, and autonomous motor engagement. This shift is especially important for adolescents with ASD, for whom alexithymia (14) and atypical interoceptive processing (15) may limit the validity of conventional self-report approaches. By translating core flow conditions into behaviorally anchored indicators (18, 19, 29), VR–Flow–BARS tool extends flow assessment to proxy-based observational contexts in adapted physical activity settings.

### 4.6 Practical implications

The VR–Flow–BARS tool is the first BARS-format proxy assessment measure designed to evaluate, in real time, the flow-related behaviors of adolescents with ASD during VR sports participation, addressing the assessment gap created by the lack of feasible self-report measurements for this population. The scale could help special education teachers and adapted physical education instructors assess students’ immersive behaviors against structured behavioral criteria and use those assessments to inform task-difficulty adjustments, feedback modifications, and individualized intervention strategies. The BARS format offers a practical advantage over abstract Likert-type scales: because concrete behavioral description statements are provided at each rating level, field practitioners can apply consistent criteria. Moreover, reinterpreting ASD-specific behavioral characteristics—peripheral gaze, stereotypic behavior, and delayed responses—as atypical indicators of flow is a systematic effort to address the inadequacy of general-population flow scales when applied to individuals with ASD.

In field applications, the VR–Flow–BARS tool may also serve as a formative assessment for monitoring progress toward adapted physical education goals in individualized education programs (IEPs). By providing a structured behavioral index of participation quality—encompassing task concentration, autonomous engagement, feedback responsiveness, and challenge persistence—the scale offers a more granular assessment of IEP goal attainment than binary participation records, and gives practitioner-level evidence for individualized adjustments to intervention parameters based on a student’s observed flow profile. However, in the absence of test–retest data, individual VR–Flow–BARS scores are best treated as session-specific behavioral snapshots. Practitioners are therefore advised to use the scale for within-session monitoring and instructional adjustment rather than to draw conclusions about stable individual differences in flow capacity.

### 4.7 Limitations

Several limitations should be acknowledged. First, although the alternative four-factor model merging FR and CEP showed substantially poorer fit than the hypothesized five-factor model, discriminant validity between the two remains borderline: the 95% bootstrap confidence interval for the FR–CEP HTMT estimate [0.851, 0.939] extended beyond the 0.90 threshold. The findings therefore support retaining FR and CEP as separate factors but do not provide definitive evidence of their discriminant validity, and their distinctiveness should be examined in independent samples.

Second, this study focused primarily on internal structural validity. External criterion-based validation—criterion-related validity, concurrent validity, and test–retest reliability—remains an important avenue for future research.

Third, rater training was conducted by video conference and written guidelines rather than in-person calibration sessions, which may have contributed to the moderate inter-rater reliability for the AME and CEP factors. Although the additional rater-level analyses indicated only modest clustering in both samples, residual rater-specific leniency or severity effects cannot be completely excluded.

Fourth, no study-specific standardized measures of ASD symptom severity, cognitive ability, or verbal functioning were available. Although participants had been formally identified as having ASD through the South Korean special education eligibility system, their functional eligibility was determined from educational records and teacher or instructor judgments rather than from standardized clinical or support-needs measures. These judgments were not independently double-rated, which may have introduced subjectivity into participant selection. Moreover, although chronological age was recorded, age-related measurement equivalence was not examined. Given the substantial heterogeneity in developmental and functional characteristics among adolescents with ASD, the sample could not be characterized or stratified by standardized ASD severity, cognitive or verbal functioning, or developmental profile. The generalizability of the findings to specific ASD subgroups and across the full adolescent age range therefore remains limited. Future validation studies should incorporate standardized functional measures and examine age-related differences alongside developmental and functional characteristics.

Fifth, the participating schools and community facilities were not randomly selected from a national sampling frame, and recruitment was non-probability based. Therefore, the samples should not be considered representative of the broader population of adolescents with ASD in South Korea, which limits the external validity of the findings. Generalization beyond adolescents with similar functional characteristics and VR participation backgrounds should be made cautiously.

Sixth, the study was limited to screen-based, non-immersive VR environments, and the applicability of the VR–Flow–BARS tool to HMD-based immersive VR contexts warrants separate investigation.

Seventh, both samples included a higher proportion of male participants (73.0% in Sample 1 and 74.0% in Sample 2). This imbalance was not intentionally imposed during recruitment and is broadly consistent with the well-documented male predominance in identified ASD populations (44). However, sex-based measurement invariance was not examined. Therefore, the findings do not establish whether VR–Flow–BARS functions equivalently across male and female adolescents with ASD, and this should be tested in future validation studies with adequately sized sex-stratified samples.

Eighth, the behavioral assessment was confined to a single observation session, so the temporal stability of the scale has not been established. Until test–retest data are available, single-session VR–Flow–BARS scores should be read as a behavioral snapshot of flow-related engagement in a given session rather than as stable trait-level indices of an individual’s flow capacity.

## 5 Conclusion

This study developed and initially validated VR–Flow–BARS tool, a proxy assessment scale for evaluating flow-related behaviors in adolescents with ASD during screen-based gamified VR sports participation. It demonstrated an acceptable factor structure, high internal consistency, adequate inter-rater reliability, and convergent and discriminant validity. As the first BARS-format instrument to systematically operationalize flow as an observable, proxy-rated behavior tailored for adolescents with ASD, the VR–Flow–BARS offers a practical, theoretically grounded measure for monitoring immersive behaviors and informing individualized intervention design in VR-based physical activity and rehabilitation programs for this population.

## Data Availability

The original contributions presented in the study are included in the article/supplementary material; further inquiries can be directed to the first author, Ahra Oh (oh-yang0329@hanmail.net).
