# Supplementary material for: Flow in screen-based gamified virtual reality sports: development and validation of a behaviorally anchored proxy rating scale for adolescents with autism spectrum disorder
Source: Front Public Health. 2026 Sep 10;14:1947117. doi: 10.3389/fpubh.2026.1947117 (PMC13600849; doi:10.3389/fpubh.2026.1947117)
Supplement: Supplementary file 1 [file Table_1.docx]

# 1 Appendix. VR–Flow–BARS Scale

**VR Sports Flow Observation Scale (VR–Flow–BARS)**

Behaviorally anchored rating scale for proxy assessment of flow in students with autism spectrum disorder

Based on Csikszentmihalyi’s flow theory adapted for observational proxy measurement

**Observation Guidelines**

| Item | Guideline |
| --- | --- |
| Observation Window | 15 min (flexible range: 10–20 min) |
| Target Phase | Main activity phase only (exclude first 3–5 min warm-up/familiarization) |
| Rating Timing | Complete immediately after observation ends |
| Avoid | Do NOT rate from memory after a 40-min full class session—observe and rate within the designated window |

**Rating Guide**

| 5—Always  ≥90% of observed time | 4—Often  70%–89% | 3—Sometimes  50%–69% | 2—Rarely  30%–49% | 1—Never  <30% |
| --- | --- | --- | --- | --- |

**① Motor Concentration**

The degree to which the student maintains continuous attention on body movement and the task during a VR sports activity

Flow dimension: Concentration on the task at hand

| Item 1 | The student maintains gaze toward the VR screen or task direction during the activity. |
| --- | --- |
| Score | Behavioral Anchor |
| 5—Always | Maintains gaze toward the screen for the vast majority of the activity (90%+). Rarely looks away even when external stimuli are present. |
| 4—Often | Maintains gaze toward the screen for most of the activity (70%–89%). Momentary distractions occur but the student returns within 2–3 s. |
| 3—Sometimes | Maintains gaze approximately half of the time (50%–69%). Intermittently looks away in response to external stimuli. |
| 2—Rarely | Maintains gaze for only a short portion of the activity (30%–49%). Looking away is more frequent than sustained attention. |
| 1—Never | Rarely or never looks at the screen (less than 30%). Gaze is consistently directed at the ceiling, floor, or surrounding people/objects. |
| Note | For students who use peripheral (sideways) gaze: assess not by gaze direction but by whether the student responds to stimuli on screen. |

| Item 2 | Despite surrounding sounds, people, or environmental stimuli, the student does not turn their gaze or body away from the screen or task. |
| --- | --- |
| Score | Behavioral Anchor |
| 5—Always | Does not redirect gaze or body toward external stimuli at all. Maintains full orientation toward the screen even in a stimulating environment. |
| 4—Often | Briefly reacts to external stimuli but returns to the task within 2–3 s without significant disruption to activity flow. |
| 3—Sometimes | Occasionally (2–3 times) redirects attention to external stimuli but generally returns to the task. |
| 2—Rarely | Redirects gaze or body toward external stimuli frequently (4+ times); takes considerable time to return to the task each time. |
| 1—Never | Repeatedly reacts to external stimuli, causing continuous disruption. Spends more time attending to the environment than to the task. |
| Note | Students with heightened sensory sensitivity may respond strongly but briefly to auditory stimuli—evaluate by the overall pattern of returning to the task. |

| Item 3 | During VR activity, task-irrelevant behaviors (e.g., leaving the activity area, sharp increase in repetitive body movements, manipulating other objects) are absent or reduced. |
| --- | --- |
| Score | Behavioral Anchor |
| 5—Always | No task-irrelevant behaviors are observed throughout. Markedly reduced compared with the student’s baseline. |
| 4—Often | Task-irrelevant behaviors appear briefly 1–2 times but cease immediately; student returns to the activity. Generally below baseline level. |
| 3—Sometimes | Task-irrelevant behaviors occur intermittently (3–4 times) but the activity continues. Frequency is similar to baseline. |
| 2—Rarely | Task-irrelevant behaviors occur frequently (5+ times), repeatedly interrupting activity flow. Above baseline level. |
| 1—Never | Persistent task-irrelevant behaviors make meaningful participation nearly impossible. |
| Note | Stereotyped movements maintained at the student’s usual baseline level are NOT penalized—only a sharp increase is relevant. Distinguish excitement-driven increases (positive) from anxiety-driven increases (negative). |

**② Task Understanding and Goal Orientation**

The degree to which the student understands the required movements and goals and performs actions accordingly

Flow dimension: Clear goals

| Item 4 | The student performs the movements required by the VR activity (e.g., reaching, turning, moving in a specific direction). |
| --- | --- |
| Score | Behavioral Anchor |
| 5—Always | Task-relevant body parts and movement types are consistently engaged. The student attempts task-appropriate movements on every trial, even if imperfect. |
| 4—Often | Task-appropriate movements are performed on most trials; occasionally an off-task movement is interspersed. |
| 3—Sometimes | Task-appropriate and task-irrelevant movements are mixed. Task-relevant movement is observed in approximately half of the trials. |
| 2—Rarely | Task-appropriate movement is rarely observed. Most movements are unrelated to the task. |
| 1—Never | No task-required movements are observed. The student holds the controller but makes no attempt at task-related actions. |
| Note | Students with motor limitations may produce small or incomplete movements—score based on directional attempt, not execution quality or success. |

| Item 5 | While moving, the student orients their gaze or body toward the goal on the VR screen (e.g., target, score display, position). |
| --- | --- |
| Score | Behavioral Anchor |
| 5—Always | Immediately orients gaze and body toward the target or goal when it appears. Directional adjustments are consistently observed as goals shift. |
| 4—Often | Orients toward goals in most instances; occasionally, the adjustment is delayed or missed. |
| 3—Sometimes | Orienting toward goals and failing to orient are equally mixed. Responds to approximately half of goal changes. |
| 2—Rarely | Rarely adjusts gaze or body toward goals when they change. Tends to look at the screen without directional tracking. |
| 1—Never | No goal-directed gaze or body orientation is observed. Maintains a fixed direction regardless of goal position. |
| Note | For students using peripheral gaze: use the subsequent movement direction to infer goal awareness. Slightly delayed orienting that ultimately aligns with the goal should receive a higher score. |

| Item 6 | The student attempts to move their body in accordance with the direction, timing, or speed required by the task. |
| --- | --- |
| Score | Behavioral Anchor |
| 5—Always | Movements consistently match the direction, timing, and speed demanded by the task. Adjusts when the target position or speed changes. |
| 4—Often | Movements largely match task requirements; one aspect (e.g., speed) occasionally deviates. |
| 3—Sometimes | Roughly matches one task parameter but frequently deviates on others. |
| 2—Rarely | Task-appropriate direction, timing, or speed is only rarely observed. Mostly repeats the same movement regardless of stimulus changes. |
| 1—Never | Movements are entirely unrelated to task direction, timing, or speed. No attempt to match task parameters is observed. |
| Note | Distinguish from Item 4 (whether movement occurs): this item assesses whether movement matches the direction, timing, and speed. Allow for slower processing—score direction match over timing precision. |

**③ VR Feedback Responsiveness**

The degree to which the student responds to visual, auditory, and score-based feedback provided in the VR environment

Flow dimension: Unambiguous feedback

| Item 7 | Following a visual cue (e.g., light flash, color change, target appearance) or auditory cue (e.g., sound effect, alert) from the VR screen, a body movement or gaze shift is observed. |
| --- | --- |
| Score | Behavioral Anchor |
| 5—Always | Consistent body movement or gaze shift is observed within 1–2 s of visual or auditory cues. The stimulus–response pattern is stable throughout. |
| 4—Often | Responds to most cues within 1–3 s; occasionally delayed or no response is observed. |
| 3—Sometimes | Responding and not responding to cues are equally mixed. A physical response is observed for approximately half of the cues. |
| 2—Rarely | Responses to cues are rare or delayed by 5+ s. Continues the same movement regardless of cue occurrence. |
| 1—Never | No body movement or gaze shift is observed in response to visual or auditory cues. The student appears unaware of the stimuli. |
| Note | Students with auditory hypersensitivity may cover their ears at sound cues—supplement judgment with responses to visual cues. For slower processors, up to 3 s may be considered within the range. |

| Item 8 | After viewing the score or result screen, the student shows a different movement (direction, speed, force, etc.) on the next attempt. |
| --- | --- |
| Score | Behavioral Anchor |
| 5—Always | Consistently adjusts the movement direction, speed, or force after viewing results. A clear pattern of repeating successful and modifying unsuccessful movements is observed. |
| 4—Often | Adjusts movement after viewing results in most instances; occasionally repeats the same movement regardless of the outcome. |
| 3—Sometimes | Adjusting and not adjusting after result feedback are equally mixed. Inconsistent use of feedback. |
| 2—Rarely | Glances at the result screen but subsequent movements rarely change. Mostly repeats the same movement regardless of outcome. |
| 1—Never | Does not look at or ignores the result screen. No movement adjustment based on results is observed. |
| Note | Repeating a successful movement is also a form of feedback utilization—distinguish from random repetition. |

| Item 9 | Following feedback (score, sound, screen response), the speed, direction, or force of the student’s movement differs from the previous attempt. |
| --- | --- |
| Score | Behavioral Anchor |
| 5—Always | A clear change in one or more movement parameters (speed, direction, force) following feedback is repeatedly observed. |
| 4—Often | Movement parameters change after most feedback; occasionally, no change is observed. |
| 3—Sometimes | Parameter changes following feedback are only intermittent. Changing and not changing occur at roughly equal frequency. |
| 2—Rarely | Changes in speed, direction, or force after feedback are rarely observed. Mostly repeats with the same intensity and direction. |
| 1—Never | Speed, direction, and force remain identical regardless of feedback content. No parameter-level change is observed. |
| Note | Distinguish from Item 8 (whether change occurs): this item focuses on the specific parameters that change (speed, direction, force). Note that overall force increase due to excitement may not reflect feedback responsiveness. |

**④ Challenge Engagement and Persistence**

The degree to which the student continues attempts even in tasks with difficulty

Flow dimension: Challenge-skill balance

| Item 10 | The student restarts movement after a failure (e.g., missing a target, game over) in the VR activity. |
| --- | --- |
| Score | Behavioral Anchor |
| 5—Always | Independently restarts the next movement within 5 s of failure, without external prompting. Consistent retry pattern is observed throughout. |
| 4—Often | Self-initiates retry after most failures; occasionally requires a brief wait or a single prompt. |
| 3—Sometimes | Retrying and stopping after failure are mixed. Occasional teacher prompting is needed to restart. |
| 2—Rarely | Mostly stops after failure; rarely retries without teacher prompting. Negative reactions to failure are frequent. |
| 1—Never | Following failure, exhibits activity-stopping behaviors (crying, leaving the area, putting down the controller) and does not retry even when prompted. |
| Note | A student who immediately restarts with a neutral expression also scores 5. Brief increase in stereotypy after failure followed by return to activity—judge by the return, not the increase. |

| Item 11 | Even in conditions of high difficulty or repeated failure, the student maintains participation without leaving the activity area or putting down the equipment. |
| --- | --- |
| Score | Behavioral Anchor |
| 5—Always | Maintains participation within the activity area, holding the controller, even through consecutive failures or increased difficulty. No leaving or putting down equipment is observed. |
| 4—Often | Mostly maintains participation; briefly pauses or looks around 1–2 times but ultimately returns to the activity. |
| 3—Sometimes | Maintaining and briefly leaving the area or putting down equipment are mixed. Returns after teacher prompting. |
| 2—Rarely | Frequently leaves the activity area or puts down the controller when difficulty increases. Requires prompting to return. |
| 1—Never | Persistently leaves the area or puts down the equipment during increased difficulty or repeated failure, refusing to continue. |
| Note | Brief pauses due to physical fatigue should not be coded as refusal. Distinguish from Item 10 (single failure retry): this item assesses overall sustained participation under accumulated difficulty. |

| Item 12 | As difficulty increases, the student continues the activity without exhibiting refusal behaviors such as crying, leaving the area, throwing the equipment, or verbal refusals. |
| --- | --- |
| Score | Behavioral Anchor |
| 5—Always | No refusal behaviors are observed when difficulty increases. Even with a tense expression, the student continues without refusal behavior. |
| 4—Often | Refusal behaviors are largely absent; brief vocalizations (e.g., “Ah!,” “Ugh!”) followed by immediate return to activity occur intermittently. |
| 3—Sometimes | Refusal behaviors occur 1–2 times but do not persist and the student returns to the activity. |
| 2—Rarely | Refusal behaviors occur frequently (3+ times) when difficulty increases, repeatedly disrupting activity flow. |
| 1—Never | Immediately upon an increase in difficulty, strong refusal behaviors appear and the activity is terminated. |
| Note | Brief exclamatory vocalizations immediately followed by returning to the activity are not coded as refusal. |

**⑤ Autonomous Motor Engagement**

The degree to which the student voluntarily initiates and sustains movement participation without external instruction

Flow dimension: Action–awareness merging / Sense of control

| Item 13 | The student initiates movement without verbal instruction, physical prompting, or gestures from the teacher or assistant. |
| --- | --- |
| Score | Behavioral Anchor |
| 5—Always | As the VR screen starts, the student independently begins movement without any prompt. Spontaneous initiation is consistently observed in every round. |
| 4—Often | Mostly initiates independently; on 1–2 occasions, a light gesture or eye contact from the teacher is needed. |
| 3—Sometimes | Independent initiation and prompt-dependent initiation are equally mixed. Approximately half of the starts occur with gesture-level cues only. |
| 2—Rarely | Verbal instruction from the teacher is needed for most starts. Independent initiation is rare. |
| 1—Never | Cannot initiate movement without physical prompting (hand-holding, arm guidance). All starts require direct physical contact. |
| Note | VR screen visual/auditory stimuli serving as natural start signals count as spontaneous initiation. If one prompt is needed initially but subsequent starts are independent, consider 4 or above. |

| Item 14 | During the activity, the student sustains participation independently without additional prompts (verbal, physical, or gestural) from the teacher or assistant. |
| --- | --- |
| Score | Behavioral Anchor |
| 5—Always | Participation is sustained continuously throughout the activity without any additional prompting. Continues even when the teacher is not present or is attending to others. |
| 4—Often | Mostly sustains participation without prompts; on 1–2 occasions, light verbal encouragement is associated with continued engagement. |
| 3—Sometimes | Sustaining without prompts and stopping are equally mixed. Intermittent verbal prompting is needed. |
| 2—Rarely | Participation requires repeated prompting to sustain. Frequently stops when prompts are absent. |
| 1—Never | Participation cannot be maintained without continuous physical prompting. Immediately stops when prompting ceases. |
| Note | Distinguish from Item 13 (initiating): this item assesses whether participation is sustained during the activity without prompts. |

| Item 15 | Beyond the movements demonstrated or instructed by the teacher, the student attempts to modify movements in their own direction, speed, or posture, or tries a new movement. |
| --- | --- |
| Score | Behavioral Anchor |
| 5—Always | Repeatedly modifies or initiates movements in their own way (different direction, speed, posture, or body part) beyond instructed movements. Spontaneous variation is prominent throughout. |
| 4—Often | Spontaneous modifications are frequently observed. Uses the teacher’s movement as a base but regularly adds personal adjustments. |
| 3—Sometimes | Spontaneous modifications appear intermittently. Imitation of instructed movements and spontaneous variation are mixed. |
| 2—Rarely | Mostly imitates the instructed movement; spontaneous modifications are rare. |
| 1—Never | Only imitates the movement demonstrated by the teacher, or no spontaneous movement attempts beyond instructions are observed. |
| Note | Avoid confusing strong imitation tendencies (characteristic of some students) with the absence of flow. Distinguish task-irrelevant behaviors from spontaneous variation within the task context. |
